# Proline Accumulation in Leaves of *Periploca sepium* via Both Biosynthesis Up-Regulation and Transport during Recovery from Severe Drought

**DOI:** 10.1371/journal.pone.0069942

**Published:** 2013-07-17

**Authors:** Yuyan An, Meixiang Zhang, Guobin Liu, Ruilian Han, Zongsuo Liang

**Affiliations:** 1 College of Life Sciences, Northwest A&F University, Yangling, Shaanxi, China; 2 Research Center of Soil and Water Conservation and Ecological Environment, Chinese Academy of Sciences, Yangling, Shaanxi, China; National Taiwan University, Taiwan

## Abstract

Drought resistance and recovery ability are two important requisites for plant adaptation to drought environments. Proline (Pro) metabolism has been a major concern in plant drought tolerance. However, roles of Pro metabolism in plant recovery ability from severe drought stress are largely unexplored. *Periploca sepium* Bunge has gained increasing attention for its adaptation to dry environments. Here, we investigated Pro metabolism in different tissues of *P*. *sepium* seedlings in the course of drought stress and recovery. We found that leaf Pro metabolism response during post-drought recovery was dependant on drought severity. Pro biosynthesis was down-regulated during recovery from -0.4 MPa but increased continually and notably during recovery from -1.0 MPa. Significant correlation between Pro concentration and Δ1-pyrroline-5-carboxylate synthetase activity indicates that Glutamate pathway is the predominant synthesis route during both drought and re-watering periods. Ornithine δ-aminotransferase activity was up-regulated significantly only during recovery from −1.0 MPa, suggesting positive contribution of ornithine pathway to improving plant recovery capacity from severe drought. In addition to up-regulation of biosynthesis, Pro transport from stems and roots also contributed to high Pro accumulation in leaves and new buds during recovery from −1.0 MPa, as indicated by the combined analysis of Pro concentration and its biosynthesis in stems, roots and new buds. Except its known roles as energy, carbon and nitrogen sources for plant rapid recovery, significant positive correlation between Pro concentration and total antioxidant activity indicates that Pro accumulation can also promote plant damage repair ability by up-regulating antioxidant activity during recovery from severe drought stress.

## Introduction

Plant responses to drought stress include changes in metabolite levels and activity of specific metabolic pathways [1]. Identification of the adaptive metabolic changes and determination of their contribution to drought resistance have long been central concerns in research on stressed plants. In many plant species, proline (Pro) accumulation is one of the main metabolic responses to abiotic stresses including high salinity, high light and UV irradiation, heavy metals and drought stress [2], [3], [4]. Due to the complexity of drought itself and plant's complex behavioral responses to drought [5], Pro metabolism in drought stressed plants is still not clearly established [6]. Furthermore, recovery ability is an important component for plant drought adaptation [7]. However, Pro metabolism in plants during recovery from severe drought and the specific effects of Pro on recovery ability have received much less attention.

For many plants, proline synthesis is activated and its degradation repressed during drought, whereas re-watering triggers the opposite regulation [4], [8]. Therefore, plant Pro accumulates to higher level during drought and recovers when re-watered [6]. This seems to be a “standard model” of plant Pro metabolism during drought and re-watering. However, exceptions have been reported [1]. For example, Vankova et al. [7] observed persistence of elevated Pro concentrations in tobacco during early stage of recovery from severe drought. Similarly, but more notably, our previous studies showed that *Periploca sepium* Bunge (*P*. *sepium*) accumulated its highest level of Pro during recovery from severe drought stress (data not published). What then is the source of Pro accumulation during re-watering period? Does Pro accumulation during recovery period depend on drought severity? Until now, little comparative research has been reported about Pro metabolism under mild and severe drought stresses. Therefore, in this study, one of our goals was to reveal the difference between Pro biosynthesis metabolism under mild and severe drought conditions.

In plants, Pro is synthesized by two pathways, the glutamate (Glu) pathway and the ornithine (Orn) pathway [4], [9]. Δ^1^-pyrroline-5-carboxylate synthetase (P5CS, EC 1.5.1.12) and ornithine δ-aminotransferase (OAT, EC 2.6.1.13) are two key enzymes involved in Pro biosynthesis [10]. Of these two anabolic pathways, Glu pathway has been generally accepted as the predominant route under stressed conditions [10]. Additionally, Pro transport has also been reported to contribute to Pro accumulation in some tissues under stressed conditions. For example, high Pro concentration in the phloem sap indicated Pro transport to growing tissues in drought-stressed alfalfa [11]. Similarly, Raymond and Smirnoff [12] presented evidence for Pro transport to the root tips of maize at low water potentials. These studies suggest that Pro transport is increased under drought-stressed conditions [9]. However, little research has been devoted to Pro accumulation mechanism and Pro transport in plants that relieved from stressed conditions.

How Pro accumulation enhances plant drought resistance is a long-standing question. In addition to the traditional roles in plant development [13], [14] and osmotic adjustment [3], Pro has also been suggested to stabilize the structure of enzymes and proteins, maintain membrane integrity and scavenge reactive oxygen species (ROS) [6], [10]. Besides, proline can also act as energy, carbon and nitrogen sources [4] or help restoration of chloroplast function [7] during recovery. Exogenous application is an efficient assistant approach to analyze roles of specific metabolites in stressed plants. Exogenous Pro has been reported to alleviate salt- and metal-induced oxidative stress in many plants [15], [16]. And its general functional mechanism is enhancing plant antioxidant enzyme activities. The high endogenous proline by transgenic method [17] has also been reported to induce high expression of several antioxidant enzymes. But to date, limited information is available on the role of actively accumulated Pro in regulating plant antioxidant activity under drought conditions. Therefore, this is the second goal of the present study.

*Periploca sepium* Bunge is a native woody vine on the Loess Plateau of China. Recently it has gained increasing attention for its adaptation to dry environments [18], [19], [20] as well as its medicinal and economical value [21], [22]. The present study was focused on elucidating Pro metabolism adjustments in hydroponic *P*. *sepium* seedlings at the whole plant level towards different degrees of water stresses and the subsequent re-watering. Besides the standard model of Pro metabolism under mild drought condition, a new model under severe drought condition was reported. In this new model, Pro accumulated substantially during the early stage of recovery. Its accumulation mechanism was then dissected and its role in plant recovery was discussed. The relationship between Pro accumulation and leaf antioxidant activity was further evaluated by principal component analysis (PCA) by exogenous Pro experiments.

## Materials and Methods

### Ethics statement

This study was conducted according to relevant national and international guidelines. Seeds collections necessary to scientific research were authorized by Ansai Research Station of Soil and Water Conservation and Ecological Environment, Chinese Academy of Sciences. Their impact upon the environment is considered negligible and these field studies do not involve endangered or protected species.

### Plant materials, growth conditions and stress imposition

In October 2011, mature seeds of *P. sepium* were collected from Gaoqiao (36°39′N, 109°11′E), a representative location in the Loess Plateau of Northwest China. Seeds were stored in a dry chamber at room temperature (15 to 20°C). In March 2012, randomly selected seeds were evenly placed in seedling culture pots (28 cm diameter and 35 cm height) filled with 13 kg of air-dried, ground and sieved (0.5 mm) substrate (3 soil: 1 wormcast). Seeds germinated after 20 days. All seedlings were grown in controlled conditions at 27 ± 2°C with a 12-h photoperiod (400 µmol·m^−2^·s^−1^ PAR). When seedlings developed shoots of 25–29 cm length and leaves of 11–13 pairs (77 days after germination), they were transferred to hydroponic pots containing half-strength Hoagland nutrient solution and thereafter allowed to acclimate for five days.

Plants were then subjected to drought stress and re-watering. Drought stress was created by using PEG-6 000. We set 0, −0.4 and −1.0 MPa as control, mild and severe drought stress, respectively. As high rates of stress could not greatly induce proline accumulation, pretreatment with lower degrees of drought stress was carried out to simulate slow-drying. Mild drought stressed-plants were pretreated with −0.2 MPa PEG 6 000 solution for two days. Severe drought stressed-plants were pretreated with −0.2 MPa PEG 6 000 solution for two days and then with −0.4 MPa PEG 6 000 solution for another two days. Exogenous Pro was applied to further analyze the effect of Pro on plant antioxidant capacity. To further determine the time effects of exogenous Pro, application of Pro during drought (+Pro) and re-watering (--Pro) periods were separately performed. Solutions were renewed every 2 days during the whole experiment. The use of −0.4 MPa, −1.0 MPa and 10 mM proline were based on the preliminary experiments in our laboratory (data not shown). Each treatment consisted of 4 pots (groups) of 30 plants each. Plants were re-watered after drought for 96 h and harvested fourth: the first two, after 24 h and 96 h drought and the second two, after 24 h and 96 h re-watering. For treatment −1.0 MPa, −1.0—Pro and −1.0+Pro, another sample, the new buds, were collected after 192 h re-watering. Measurements and analysis were taken on leaves (fully expanded leaves) or new buds, stem apexes (0-to 12-mm section) and roots. Each sample was divided into two groups. One group was frozen in liquid nitrogen, and stored at −80°C for enzyme activities analysis. The other one was oven dried at 105°C for 20 min and 60°C for 72 h and then ground for estimating Pro, total free amino acids (TFAA), anthocyanidin (An), total flavonoids (TF) and total phenols (TP) concentrations.

### Determination of amino acids concentration, P5CS and OAT activity

Relative water content (RWC), concentration of Pro and TFAA were determined according to An et al.[20]. P5CS extraction and assay was following the method of Sunkar [23] with slight modification. Random 0.2 gram fresh samples was homogenized with 2 mL 0.05 M pH 7.5 Tris-HCl buffer containing 1 mM EDTA, 5 mM MgCl_2_, 10 mM dithiothreitol (DTT), 1× protease inhibitor cocktail and 10 mM 3-(N-morpholino) propanesulfonic acid (MOPS). The homogenate is centrifuged at 12 000×g for 20 min at 4°C. The reaction mixture (1 mL) was consisted of 0.5 mL 50 mM pH 7.5 Tris-HCl including 1 mM EDTA, 50 mM KCl, 3 mM MgSO_4_ and 10 mM MOPS, 50 µL 200 mM DTT, 25 µL 20 mM NADPH, 60 µL 500 mM Glu, 315 µL tissue extract and 50 µL 50 mM ATP. After incubation at 25°C for 20 min, added 3.4 mL ultrapure water, 0.4 mL ammonium molybdate - sulfuric acid solution, 0.1 mL 0.1% malachite green and 0.1 mL 0.1% polyvinyl alcohol, mixed well and incubated for another 20 min. The Pi released from P5CS activity was measured at 650 nm. Enzyme activity was expressed as µg Pi g^−1^ FW h^−1^. OAT activity was assayed according to Yang et al. [24]. The absorbance of 0.01 at 510 nm was defined as one unit of OAT activity, and the enzyme activity was expressed as unit g^−1^ FW h^−1^.

### Assay of antioxidant enzyme activities

Random 0.1 gram fresh samples was homogenized with 8 mL 0.05 M Na phosphate buffer solution (pH 7.8) including 1% PVP and 0.1 mM EDTA, and centrifuged at 12 000 × *g* for 20 min at 4°C. Superoxide dismutase (SOD, EC1.15.1.1), catalase (CAT, EC1.11.1.6) and ascorbate peroxidase (APX, EC1.11.1.1) were determined according to Zhu et al. [25]. One unit of SOD activity was defined as the quantity of SOD required to produce a 50% inhibition of NBT. And SOD activity was expressed as unit g^−1^ FW h^−1^. One unit of CAT was defined as decreasing 0.1 in absorbance at 240 nm in 1 min. The APX assay depended on the decrease in absorbance at 290 nm as ascorbate was oxidized. APX activity was expressed in terms of millimole ascorbate oxidized g^−1^ FW min^−1^. Peroxidase (POD, EC1.11.1.7) activity and SP concentration were specifically quantified according to An and Liang [18]. One unit of POD activity was defined as the amount of enzyme that made A470 increase 0.1 per min. The enzyme activity was expressed in terms of unit g^−1^ FW min^−1^. Glutathione reductase (GR, EC1.6.4.2) activity was assayed according to Smith et al. [26] with slight modification. 75 µL of tissue extract was used in the assay along with 1.5 mL 0.05 M Na phosphate buffer solution (pH 7.8), 0.75 mL 3 mM 5,5′-dithio-bis-(2-nitrobenzoic acid), 0.15 mL 2 mM NADPH, and 0.15 mL 20 mM oxidized glutathione (GSSG). GSSG was added last to initiate the reaction and the increase in absorbance at 412 nm was recorded for 3 min. The extinction coefficient of the product 2-nitro-5-thiobenzoic acid (TNB) (14.15 M^−1^cm^−1^) was used to calculate the activity of GR which was expressed in terms of millimole TNB g^−1^ FW min^−1^. These indicators were all determined using a spectrophotometer (UV-2802H, UNICO).

### Determination of An, TF and TP concentration

Random 0.05 gram fine powdered material was suspended in 5 mL 1% HCl methanol (v/v), incubated in darkness for 24 h at 25°C and then filtered. The supernatant was used for An, TF and TP assays. For An concentration, the absorbance of diluted extract was measured at 530 nm and one unit of An was defined as increasing 0.1 in absorbance at 530 nm. An was expressed as absorbance units g^−1^ DW. TP were determined with Folin-Ciocalteu reagent by the method of Singleton et al. [27] using gallic acid as a standard. TP concentration was expressed as mg gallic acid equivalent g^−1^ DW. The aluminium chloride (AlCl_3_) method [28] was used for determination of TF concentration. The absorbance of TF (extracted with 5% NaNO_2_, 10% AlCl_3_ and 1 M NaOH) was measured at 510 nm using rutin as a standard. TF was expressed as mg rutin equivalent g^−1^ DW.

### Statistical analysis

Pot cultures were carried out in completely randomized design. Statistical analysis was performed using SPSS statistical computer package (version 16.0 SPSS Inc. Chicago. IL). Mean and standard error (SD) values of three replicates were calculated. Data was compared with the control or among treatments by analysis of variance (ANOVA) to discriminate significant differences at *P* ≤ 0.05 followed by least significant difference tests (LSD). Correlation analysis was carried out between leaf Pro concentration and its synthesis enzyme activities or total antioxidant activity. Principle component analysis (PCA) was performed using all ten antioxidant variables in order to comprehensively evaluate plant antioxidant capacity among treatments. Before the PCA analysis, the variables were standardized following a transformation by Z-score. Results were displayed using the principal component (PC) score and loading plots. The final score representing plant total antioxidant activity of each sample was calculated by the following formula: Total score = *a*_1_×*F*_1_ +*a*_2_×*F*_2_ +*a*_3_×*F*_3_, where *a*_i_ is the contribution of principal component i to the total variability and *F*_i_ is the score on principal component i.

## Results

### Leaf RWC and morphological changes in response to drought and re-watering

**Figure 1:**
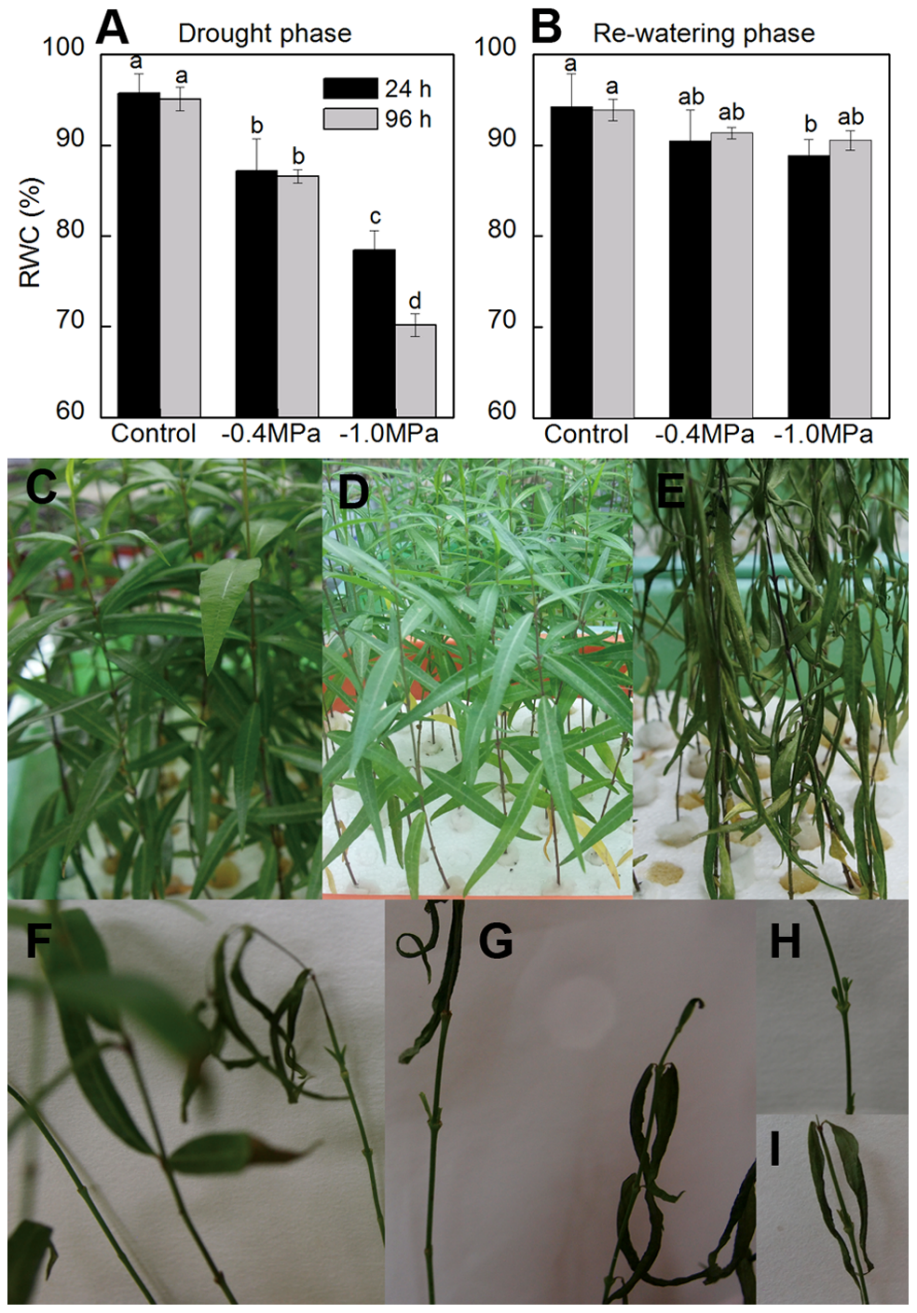
Leaf RWC and morphology in *P. sepium* seedlings during drought stress and re-watering. Two different degrees of drought treatment, −0.4 and −1.0 MPa, were set. Plants were re-watered after being drought-stressed for 96 h under −0.4 MPa or −1.0 MPa. RWC were determined after 24 and 96 hours of Control, drought stress or re-watering. (RWC) relative water content, (A, B) RWC during drought and re-watering phase, data represent the mean ± standard deviation (SD) of three replicates. Different letters at the top of each bar indicate significant differences at *P*<0.01; (C-E) seedling after 96 hours of Control, −0.4 MPa and −1.0 MPa; (F-I) new buds after 96 hours of recovery from −1.0 MPa.

To estimate the effect of drought and re-watering, leaf RWC and morphological characteristics were monitored during the whole experiment (Figure 1). Results showed that osmotic stress in our study significantly decreased leaf RWC in *P. sepium* (Figure 1A). Compared with the control, −0.4 MPa for 96 h resulted in a decline of 8.53%, while −1.0 MPa caused a decrease of 24.87%. No obvious changes were observed on leaf morphology after 96 h drought under −0.4 MPa, while 60% of leaves were accelerated aging under −1.0 MPa (Figure 1C-E). When stress was relieved, RWC of −0.4 MPa stressed plants recovered to the control level within 24 h, while that of −1.0 MPa stressed plants did in 96 h (Figure 1B). For plants relieved from −1.0 MPa, many new buds appeared along the stems on the third day of re-watering (Figure 1F-I).

### Leaf Pro biosynthesis under drought stress and during subsequent re-watering

**Figure 2:**
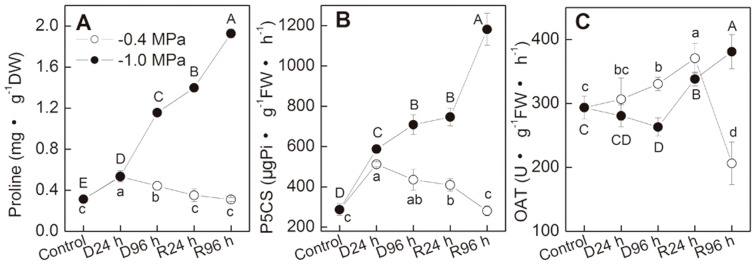
Leaf proline biosynthesis metabolism in *P. sepium* seedlings during drought stress and re-watering. Two different degrees of drought treatment, −0.4 and −1.0 MPa, were set. Plants were re-watered after being drought-stressed for 96 h under −0.4 MPa or −1.0 MPa. Parameters were determined after 0, 24 and 96 hours of drought stress or re-watering. (A) proline concentration; (B) activity of Δ^1^-pyrroline-5-carboxylate synthetase (P5CS); (C) activity of ornithine δ-aminotransferase (OAT). Data represent the mean ± standard deviation (SD) of three replicates. Different small letters along line of -0.4 MPa and capital letters along line of −1.0 MPa indicate significant differences between different time points at *P*<0.01, respectively.

Leaf Pro concentration increased significantly under −0.4 MPa, and declined to the control level when re-watered (Figure 2A). Stress of −1.0 MPa led to much higher Pro accumulation, about 1.71 and 3.69 fold of control levels in 24 h and 96 h, respectively. Surprisingly, Pro concentration in plants recovering from −1.0 MPa did not declined, but continued to increase greatly. After 96 h recovery, Pro concentration increased to 6.15 times of the control level.

To interpret the above different responses of Pro in plants to −0.4, −1.0 MPa and subsequent re-watering, activities of P5CS and OAT were analyzed at all time points (Figure 2B and C). P5CS activity showed similar trend to Pro concentration (Figure 2B). Correlation analysis showed significant correlation (−0.4 MPa, *r* = 0.931, *P = *0.021; −1.0 MPa, *r* = 0.949, *P = *0.014) between Pro concentration and P5CS activity during drought and re-watering phases. Meanwhile, OAT activity increased under −0.4 MPa while decreased under −1.0 MPa (Figure 2C). After re-watered from −1.0 MPa, OAT activity increased significantly (Pro and OAT, *r* = 0.907, *P = *0.277).

Protein degradation is one of the possible sources for Pro accumulation [29]. To explore the potential contribution of protein degradation to Pro accumulation, correlation analysis between protein and Pro concentrations were performed. Significant positive correlation (*r* = 0.889, *P = *0.044) between protein and Pro were found during mild drought (−0.4 MPa) and re-watering condition, while no obvious correlation (*r* = −0.150, *P = *0.809) between them was observed during severe drought (−1.0 MPa) and re-watering condition. This result indicated that protein degradation was not the main source of Pro accumulation in leaves of *P. sepium* during drought and re-watering conditions.

### Pro transport from stems and roots to leaves and new buds during recovery from severe drought stress

**Figure 3:**
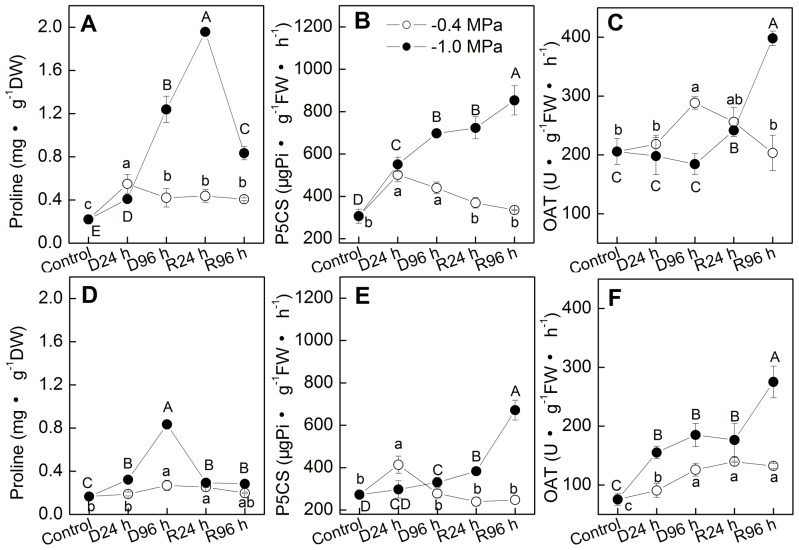
Stem and root proline biosynthesis metabolism in *P. sepium* seedlings during drought stress and re-watering. Two different degrees of drought treatment, −0.4 and −1.0 MPa, were set. Plants were re-watered after being drought-stressed for 96 h under −0.4 MPa or −1.0 MPa. Parameters were determined after 0, 24 and 96 hours of drought stress or re-watering. (A, D) proline concentration in stems and roots; (B, E) activity of Δ^1^-pyrroline-5-carboxylate synthetase (P5CS) in stems and roots; (C, F) activity of ornithine δ-aminotransferase (OAT) in stems and roots. Data represent the mean ± standard deviation (SD) of three replicates. Different small letters along line of −0.4 MPa and capital letters along line of −1.0 MPa indicate significant differences between different time points at *P*<0.01, respectively.

In addition to up-regulation of synthesis, Pro accumulation in leaves might also come from down-regulation of degradation or Pro transport from stems and roots. To investigate whether it come from transport, we simultaneously analyzed Pro accumulation, P5CS and OAT activities in stems and roots (Figure 3). Obviously, P5CS and OAT activities in both stems and roots were up-regulated significantly during plant recovery from −1.0 MPa (Figure 3B, C, E and F), while their Pro concentration declined (Figure 3A and D). This phenomenon was not observed in plants that recovered from −0.4 MPa. These results indicated that the Pro synthesized in stems and roots is likely transported to leaves during plant recovery from severe drought. To verify this point, Pro concentration in the new buds that newly germinated during re-watering was assayed (Table 1). Compared with the control, the buds showed the same level of P5CS, lower OAT activity but significantly higher Pro concentration. This result indicated that the Pro accumulation in the new buds partially come from Pro transport from other organs. To further demonstrate Pro transport, we exogenously supplied Pro to the recovery solution. Results showed that exogenous supply of Pro to the roots of seedlings led to remarkable increase in Pro concentration of new buds (6.45 times of the control). Meanwhile P5CS and OAT activities were not so greatly affected. Altogether, these results indicated Pro transport from stems and roots to leaves and new buds in *P. sepium* during recovery from severe drought. The new buds with higher Pro accumulation showed larger SP content (Table 1). This result again implied that Pro accumulation in *P. sepium* was not due to protein degradation.

**Table 1:** Proline concentration, Δ^1^-pyrroline-5-carboxylate synthetase (P5CS) activity, ornithine δ-aminotransferase (OAT) activity and soluble protein (SP) concentration in the new buds germinated during recovery from −1.0 MPa.

	Proline (mg·g^−1^DW)	P5CS ( µgPi·g^−1^FW·h^−1^)	OAT (U·g^−1^FW·h^−1^)	SP (mg·g^−1^FW)
Control	0.31 ± 0.01 C	286.81 ± 28.45 B	293.56 ± 17.86 A	9.89 ± 0.35 C
New buds ^1^	0.62 ± 0.08 B	357.81 ± 34.12 B	199.60 ± 15.41 B	15.91 ± 1.74 B
New buds ^2^	2.00 ± 0.18 A	646.05 ± 59.88 A	118.73 ± 17.25 C	20.32 ± 1.54 A

Note: Control, normal mature leaves; ^1^ new buds without proline application in recovery solution; ^2^ new buds with 10 mM proline in recovery solution; different letters in the same column indicate significant difference at *P*<0.01.

### Positive relationship between Pro accumulation and plant antioxidant capacity

**Figure 4:**
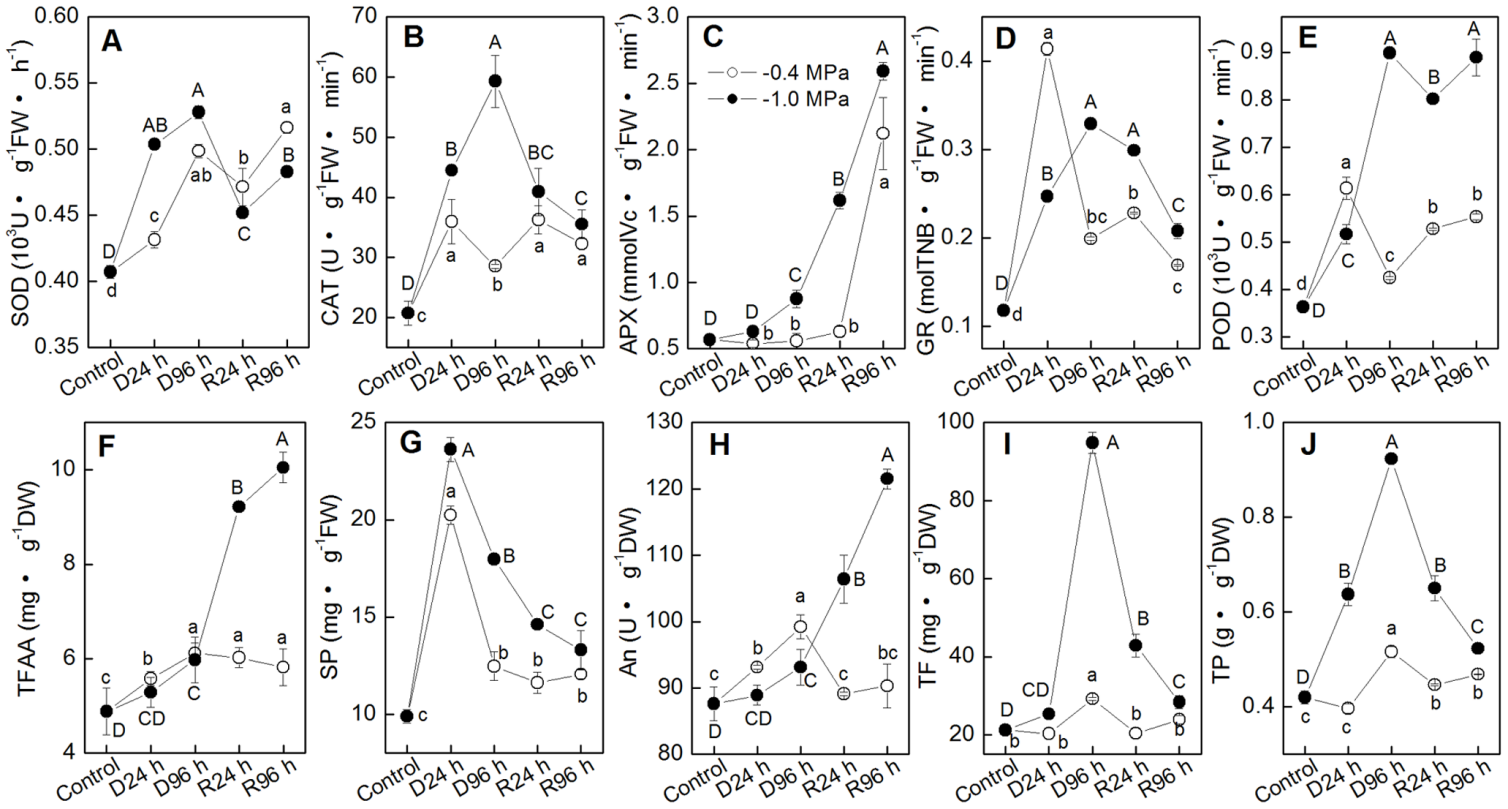
Leaf antioxidant defense in *P. sepium* seedlings during drought stress and re-watering. Two different degrees of drought treatment, −0.4 and −1.0 MPa, were set. Plants were re-watered after being drought-stressed for 96 h under −0.4 MPa or −1.0 MPa. Parameters were determined after 0, 24 and 96 hours of drought stress or re-watering. (A-E) antioxidant enzyme acitivity; (F-J) antioxidant concentration; (A) superoxide dismutase (SOD), (B) catalase (CAT), (C) ascorbate peroxidase (APX), (D) glutathione reductase (GR), (E) peroxidase (POD), (F) total free amino acids (TFAA), (G) soluble sugars (SP), (H) anthocyanin (An), (I) total flavonoids (TF), (J) total phenols (TP). Data represent the mean ± standard deviation (SD) of three replicates. Different small letters along line of −0.4 MPa and capital letters along line of −1.0 MPa indicate significant differences between different time points at *P*<0.01, respectively.

As mentioned in the introduction, both exogenous Pro and high endogenous Pro could alleviate salt- and metal-induced oxidative stress by enhancing plant antioxidant defense capacity [15], [16], [17]. Here, five antioxidant enzymes and five non-enzymatic antioxidants were determined to analyze the relationship between the active Pro accumulation and antioxidant capacity. Under −0.4 MPa, CAT, GR, POD activity and SP concentration showed strong correlation with Pro concentration (r>0.85) (Figure 4 and Table 2). Upon re-watering, TFAA, An and TP concentration displayed strong correlation with Pro concentration (r>0.81) (Table 2). Under severe drought (−1.0 MPa), all enzyme activities and antioxidants concentrations except SP increased largely with stress time prolonged (Figure 4). They exhibited substantial positive correlation with Pro concentration. During recovery from −1.0 MPa, APX activity and TFAA, An concentration increased abruptly. The correlation coefficients between Pro and each of them were all above 0.85. The enzyme activities and antioxidants concentrations under −1.0 MPa were notably higher than that under −0.4 MPa, as did Pro concentration. These results together indicated that the accumulated Pro might involve in inducing plant antioxidant defense system. The responsive protective enzymes and antioxidants seemed to depend on drought severity and treatment period (drought or re-watering period).

**Table 2:** Correlation coefficients between Pro and ten antioxidant parameters.

Treatment	Period	SOD	CAT	APX	GR	POD	TFAA	SP	An	TF	TP
−0.4 MPa	Drought	0.329	0.996	−0.956	0.937	0.854	0.644	0.910	0.561	0.000	−0.180
	Re-watering	0.060	−0.540	−0.762	0.305	−0.990	0.910	0.423	0.872	0.673	0.813
−1.0 MPa	Drought	0.827	0.921	0.997	0.924	1.000	0.993	0.352	1.000	0.979	0.982
	Re-watering	−0.332	−0.869	0.991	−0.998	0.122	0.857	−0.896	0.985	−0.864	−0.913

Note: (SOD) superoxide dismutase, (CAT) catalase, (APX) ascorbate peroxidase, (GR) glutathione reductase, (POD) peroxidase, (TFAA) total free amino acids, (SP) soluble sugars, (An), anthocyanin, (TF) total flavonoids, (TP) total phenols.

**Figure 5:**
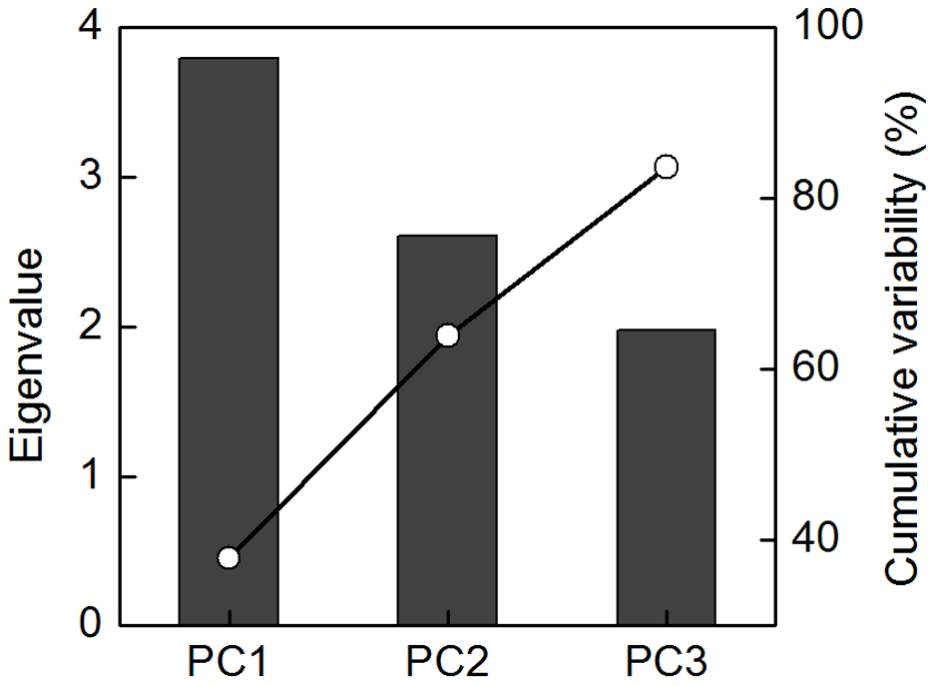
Scree plot of variance explained by each factor of the principal component. (PC1-PC3) the first, second and third principal component.

To further confirm the role of accumulated Pro in enhancing plant antioxidant activity, supplement of exogenous Pro was performed to change Pro accumulation, and then the responses of plant antioxidant defense system were analyzed. To obtain a broad view on the antioxidant capacity, the whole data set was subjected to PCA. Three principal components (PC1, PC2 and PC3) were extracted, and together these dimensions explained over 83% of the total variability (Figure 5). The data dimensions were therefore reduced from ten to three for further data processing.

**Figure 6:**
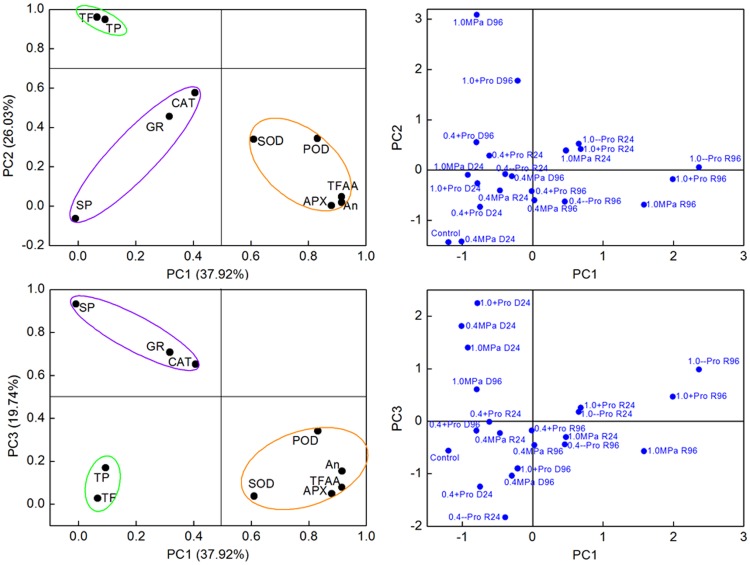
Principal component analysis (PCA) of *P. sepium* leaf samples at all time points of drought and re-watering periods. (A, C) PC1-PC2 and PC1-PC3 variables loading plots; (B, D) PC1-PC2 and PC1-PC3 samples score plots. (PC1-PC3) the first, second and third principal component; (0.4) −0.4 MPa, (1.0) −1.0 MPa, (+Pro) with 10 mM proline in stress solution, (--Pro) with 10 mM proline in recovery solution; (D24, D96) drought for 24 and 96 hours, (R24, R96) re-watering for 24 and 96 hours after 96 h drought.

PC1 was heavily and positively associated with SOD, APX, POD, TFAA and An, and PC2 gave a high weighting to TF and TP (Figure 6A). PC1 appeared to separate re-watered samples from their corresponding stressed-samples, which was especially true for severe drought conditions. These results suggested that SOD, APX, POD, TFAA and An contribute a lot in antioxidant and repair capacity of *P. sepium* from drought conditions. Distances along PC1 score between treatments with and without Pro revealed that supply of Pro promotes antioxidant activity of *P. sepium* during recovery from severe drought (−1.0 MPa). Similar effect of exogenous Pro was not obvious in plants recovered from mild drought (−0.4 MPa). PC2 highlighted accumulation of secondary antioxidants. Higher Scores of exogenous Pro treated samples in PC2 during re-watering periods indicated that Pro also promotes accumulation of TF and TP in *P. sepium* during recovery periods. This effect of exogenous Pro was particularly notable in *P. sepium* from severe drought. These results indicated that exogenous Pro could induce higher enzyme activities and antioxidants accumulation to help *P. sepium* recover from severe drought conditions. The quantitative traits that contributed more positively to PC3 included CAT, GR and SP. Distances in PC3 among re-watered samples indicated that CAT, GR and SP should be also involved in Pro-induced up-regulation of antioxidant activity during recovery from stress.

Samples during drought periods were mainly separated by PC2 and PC3. The distance of stressed samples along PC2 and PC3 demonstrated that exogenous Pro effect on plant antioxidant activity during drought periods was dependent on both drought severity and duration time. For example, exogenous Pro significantly increased accumulation of TF and TP under −0.4 MPa, but not under −1.0 MPa. As well, CAT, GR activities and SP concentration were increased by short term (24 h) exogenous Pro under −0.4 MPa and decreased under −1.0 MPa. While long term exogenous Pro functioned oppositely.

**Figure 7:**
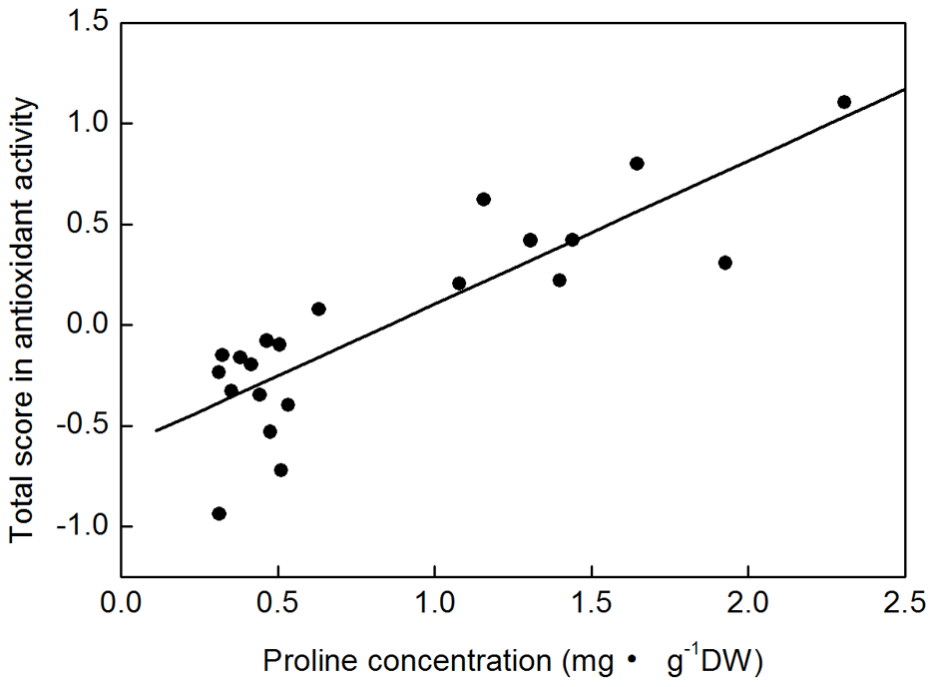
Significant positive correlation between leaf proline concentration and total antioxidant activity.

PC1, PC2 and PC3 reflected different aspects of antioxidant activity. To display the effect of exogenous Pro more intuitively, score in total antioxidant activity of each sample was calculated (Table 3). Control sample showed the lowest antioxidant activity (−0.937). Table 3 again showed that short-term Pro (24 h) led to substantial decrease under −0.4 MPa but strong increase under −1.0 MPa in antioxidant activity of *P. sepium*. However, prolonged stress (96 h) exactly reversed the Pro effect. Application of Pro during drought period also enhanced the antioxidant activity of *P. sepium* during the subsequent re-watering. This effect was especially noticeable under severe drought conditions. To further determine the time effects of exogenous Pro, application of Pro during re-watering periods was performed. Results showed that its effect was worse than that of drought-period-supplied Pro for mild drought treatments but much better for the severe ones. The total scores altogether suggested drought period and recovery period, respectively, as the better application time of Pro under mild and severe drought stress conditions to improve plant antioxidant activity. To define role of accumulated Pro in plant antioxidant activity, correlation analysis between leaf endogenous Pro concentration and total antioxidant activity was conducted (Figure 7). The significant correlation between them (*r* = 0.856, *P*<0.001) further suggested the significant positive role of the actively accumulated Pro in inducing stronger antioxidant activity.

**Table 3:** Total scores in leaf antioxidant activity of different samples.

Treatments	No Pro	+ Pro	Treatments	No Pro	+ Pro during D	+ Pro during R
−0.4 MPa D24	−0.396	−0.720	−0.4 MPa R24	−0.327	−0.162	−0.530
−0.4 MPa D96	−0.347	−0.195	−0.4 MPa R96	−0.235	−0.149	−0.077
−1.0 MPa D24	−0.098	0.078	−1.0 MPa R24	0.220	0.419	0.420
−1.0 MPa D96	0.622	0.204	−1.0 MPa R96	0.308	0.799	1.105

Note: Total scores of antioxidant activity were evaluated by principal component analysis (PCA) using ten antioxidant parameters. (+Pro) with 10 mM proline in stress solution; (D) drought, (R) re-watering, (D24, D96) drought for 24 and 96 hours, (R24, R96) re-watering for 24 and 96 hours after 96 h drought.

## Discussion

### Pro accumulation in leaves during recovery from severe drought results from up-regulation of both Glu and Orn synthesis pathways

Plant drought response is complex. It depends not only on the species inherent “strategy” but also on drought severity and stress duration time [30]. *P. sepium* is a native shrub in drought-prone regions. The present study demonstrates that response of Pro biosynthesis in *P. sepium* to drought and re-watering is strictly associated with the stress severity.

Under mild drought conditions, similar to many other reports [31], [32], Pro biosynthesis was up-regulated under drought and down-regulated during recovery period (Figure 2). As Pro did not accumulate highly enough to explain its contribution to osmotic adjustment, Pro mainly functions as a stabilizer of subcellular structures or a redox-buffering agent [10]. Under severe drought stresses, Pro accumulated to a much higher level (Figure 2), indicating that it might act as an osmoprotectant under severe stress [33], [34]. In contrast to its down-regulation during recovery from mild drought, Pro concentration increased continually and notably during recovery from the severe drought (Figure 2). These results demonstrate that Pro metabolism responses to drought and re-watering are strictly associated with drought severity. The high Pro accumulation during recovery from severe drought is consistent with a previous study in our laboratory (data not published) in which the Pro concentration then declined rapidly with re-growth of the new buds. These results suggest important roles of Pro in plant recovery from severe drought.

In addition to the roles of Pro under various stresses, increasing evidences reveal that Pro is involved in plant development, especially in flowering and reproduction [13], [14]. Not only Pro accumulation but also Pro metabolism plays essential roles in plants. On one hand, Pro can be rapidly utilized as proteinogenic amino acids and a source of carbon and nitrogen [4]. On the other hand, active catabolism of Pro promotes activities of some metabolic pathways, such as respiration [1] and restoration of chloroplast function [7]. Besides, Pro accumulation can also act as a signaling molecule to modulate mitochondrial functions, influence cell proliferation and trigger specific gene expression [4]. Therefore, the high accumulation of Pro at early stage of re-watering must be an important mechanism for rapid recovery from severe drought. Further studies on other drought tolerant plants which can survive from severe/extreme drought should be carried out to determine whether the high accumulation of Pro during recovery from severe drought is a common phenomenon.

The significant correlation between P5CS activity and Pro concentration in our study confirms that the Glu pathway is the predominant synthesis route in drought stressed plants [35]. The significance of Orn pathway and OAT in proline biosynthesis has been controversial [4]. In this study, the significant increase in OAT activity during recovery from −1.0 MPa indicates that the Orn pathway contributes to the Pro accumulation too. Recently, You et al. [36] prove that an ornithine δ-aminotransferase gene *OsOAT* confers drought and oxidative stress tolerance in rice. Our results suggest a positive role of Orn pathway in improving *P. sepium* recovery ability from severe drought stress.

Except the *de novo* synthesis, protein degradation under stress conditions may also contribute to Pro accumulation [29]. However, several studies suggested that protein degradation contributed little to Pro accumulation in plants under salt [37] and osmotic stress [38] conditions. Similar to these studies, here, no significant negative correlation was found between Pro accumulation and SP concentration in *P. sepium* under drought and re-watering conditions. This result indicates that protein degradation is not the main source of Pro accumulation in leaves of *P. sepium*. The larger SP content in the new buds with higher Pro accumulation (Table 1) further confirms that Pro accumulation in *P. sepium* is not derived from protein degradation. Under stress conditions, membrane integrity must be maintained to prevent protein denaturation. Our results support that the accumulated Pro might interact with proteins to preserve protein structure and enzyme activities under water deficit conditions [9]. The higher protein content in the new buds should be the result of enhanced protein synthesis. Therefore, one important role of the highly accumulated Pro is to accelerate protein biosynthesis, ultimately leading to rapid plant recovery. This finding agrees well with Pro's role as rapid source of carbon and nitrogen during recovery period [4].

### Pro transport from stems and roots also contributes to Pro accumulation in leaves and new buds during recovery from severe drought

Long distance transport of Pro through phloem vessels has been documented [11], [12], and some Pro transporters have been identified [39]. These studies indicate that Pro is transported from leaves to roots or meristematic tissues under water stress to improve drought tolerance. In the present study, we demonstrate convincingly Pro transport in *P. sepium* during re-watering period from severe drought. The significant up-regulation of P5CS and OAT in stems and roots was not accompanied by accumulation of Pro (Figure 3). The new buds that showed lower ability of Pro biosynthesis exhibited marked Pro accumulation (Table 1). And the Pro concentration in new buds was further considerably increased by exogenous Pro that applied through rooting medium (Table 1). These results together prove that Pro is transported from stems and roots to leaves and new buds during recovery from severe drought.

About 60% of leaves in *P. sepium* seedlings were senescent and dead (Figure 1E) after 96 h severe drought stress. The accelerating senescence has been suggested as an active re-organization process to extend plant life during severe drought [40]. Severe drought stresses always lead to remobilization of nutrients to the storage organs, stems and roots [8], [40], [41]. Therefore, *P. sepium* plants sacrifice less important organs (leaves) for plant survival in exchange for the whole plant life. However, when re-watered, as the primary organ for photosynthesis and transpiration, leaves are extremely important for plant recovery [42]. Therefore, increasing allocation of carbon and nutrients to shoots is required to improve growth rate of leaves. So, Pro transport to leaves and new buds in *P. sepium* is the result of fine turn of plant responses at the whole plant level. The higher accumulation of Pro in leaves and new buds during re-watering is mainly acting as sources of energy, carbon and nitrogen to increase the re-growth rate. It seems that stems and roots turn into source organs during early stage of recovery from long term severe drought, while new buds become the most important sinks. Over all, Pro transport among organs must be an important strategy of *P. sepium* for rapid recovery from severe drought environments.

### Plant Pro accumulation promotes not only antioxidant activity during stress but also repair ability during recovery from severe stress

Pro dependent scavenging of hydroxyl radicals [43] and singlet oxygen [44] has been reported. Kaul et al. [45] further demonstrated the free radical scavenging potential of Pro in vitro assay systems. Recently, however, Signorelli et al. [46] clearly proved that Pro could not quench singlet oxygen under stress and suggested rethinking Pro role in scavenging of hydroxyl radicals. These studies indicate controversial role of Pro in direct scavenging ROS which need further investigation. In addition to its direct role, indirect roles of Pro in scavenging ROS by enhancing plant antioxidant defense systems have also been reported [15], [16], [17]. In the current study, the increasing antioxidant activity of *P. sepium* seedlings is closely positive correlated to Pro accumulation (Figure 7). Therefore, our results provide new evidence that the actively accumulated Pro in drought stressed plants could also enhance plant antioxidant activity. Recovery ability from stress is as important as drought resistance during stress for plant survival and growth [7]. Our study, to our knowledge, first demonstrate that the highly accumulated Pro during re-watering period plays important roles in enhancing plant repair ability from drought induced oxidative stress.

As we all know, plant cells are well equipped with many antioxidant enzymes including superoxide dismutase (SOD), catalase (CAT), peroxidase (POD), ascorbate peroxidase (APX) and glutathione reductase (GR) as well as lots of non-enzymatic antioxidants such as ascorbic acid, An and TF [47], [48], [49]. The cooperation (and/or compensation) of different antioxidant systems plays a pivotal role in the competence of plant antioxidant defense system [47]. Our data show that, in addition to enhancing activities of antioxidant enzymes [15], [16], high accumulation of Pro can also promote production of many non-enzymatic antioxidants, such as An, TF and TP (Figure 4 and 6). Drought-stressed samples were separated from each other by PC2 and PC3 (Figure 6). And PC2 and PC3 highlight CAT and GR activities, SP, TF and TP concentrations. These data indicate that Pro-induced changes in antioxidant activity during drought stresses are mainly mediated by CAT, GR, SP, TF and TP. Secondary metabolites play important roles in plant adaptation to the changing environment and in overcoming stress conditions [48], [50]. Similar to the study on maize (*Zea mays* L.) [51], exogenous Pro increased accumulation of secondary TF and TP under drought stress, confirming that Pro might also involve in activation of plant secondary metabolism [52]. When re-watered, plants with higher Pro concentration showed stronger antioxidant activity. Nounjan et al. [15] reported that exogenous Pro increased APX activity and promoted a stronger ability of plants to recover from salt stress. Here, distances of re-watered plants along PC1, PC2 and PC3 demonstrate that highly accumulated Pro increased activities of all five antioxidant enzymes and accumulations of all antioxidants. Therefore, in addition to its roles as sources of energy, carbon and nitrogen [4], [53], our result suggests a regulatory/signaling role of highly accumulated Pro in promoting plant damage repair ability during recovery from severe drought. This result provides useful information on attractive tool with which to promote plant recovery ability from severe drought stress. This is crucially important for improving plant performance during drought environments especially in consideration of global warming.

## Conclusions

Different plant species are highly variable with respect to their optimum environments, drought resistance and stress-response mechanisms [8]. This study about *P. sepium*, a drought-resistant species, adds new information on plant Pro biosynthesis metabolism, Pro transport and Pro roles in plant recovery from drought. We proposed a new response model of Pro biosynthesis in *P. sepium*. In this model Pro accumulates remarkably in leaves and new buds during the early stage of recovery from prolonged severe drought stress. And its accumulation is proved to be the combined result of biosynthesis up-regulation and Pro transport from stems and roots. In addition to its known roles in improving plant tolerance during drought period or acting as energy, carbon and nitrogen sources for plant recovery, highly accumulated Pro can also promote plant damage repair ability by up-regulating antioxidant activity during recovery from severe drought stresses. A number of studies have used exogenous application or transgenic strategies to modify Pro metabolism in order to enhance drought resistance. Our new model suggests that, modifications of Pro metabolism during recovery, such as enhancing Pro synthesis coupled with increasing Pro transport to leaves or new buds, could be an effective strategy to promote plant recovery ability from severe drought.
